# Combination of the Distance From Tumor Edge to Subventricular Zone and IDH Mutation Predicts Prognosis of Patients With Glioma

**DOI:** 10.3389/fonc.2021.693693

**Published:** 2021-08-19

**Authors:** Shuixian Zhang, Fengchun Zhao, Tengyuan Zhou, Dan Liu, Xiaohong Yao, Wenjuan Fu, Zhi Liu, Chuan Lan, Zhaopan Lai, Chen Liu, Haitao Li, Yuhong Li, Shengli Hu, Yi Yin, Liang Tan, Wenyan Li, Fei Li, Rong Hu, Hua Feng

**Affiliations:** ^1^Department of Neurosurgery, Southwest Hospital, Third Military Medical University (Army Medical University), Chongqing, China; ^2^Department of Pathology, Southwest Hospital, Third Military Medical University (Army Medical University), Chongqing, China; ^3^Department of Radiology, Southwest Hospital, Third Military Medical University (Army Medical University), Chongqing, China

**Keywords:** glioma, MRI, subventricular zone, isocitrate dehydrogenase 1, prognosis

## Abstract

Both subventricular zone (SVZ) contact and isocitrate dehydrogenase 1 (IDH1) mutation have been reported to be related to the outcome of glioma, respectively. However, far too little attention has been paid to the role of tumor edge-SVZ distance in the outcome of glioma. We aim to assess the value of tumor-SVZ distance, as well as combined tumor-SVZ distance and IDH status, in predicting the outcome of gliomas (WHO grade II–IV). Here, the MR images and clinical data from 146 patients were included in the current study. The relationship between survival and the tumor-SVZ distance as well as survival and combination of tumor-SVZ distance and IDH status were determined *via* univariate and multivariate analyses. In univariate analysis of tumor-SVZ distance, the patients were divided into three types (SVZ involvement, tumor-SVZ distance from 0 to 10 mm, and tumor-SVZ distance >10 mm). The results showed that the OS (p = 0.02) and PFS (p = 0.002) for the patients had a positive correlation with the tumor-SVZ distance. In addition, simple linear correlation found a significant relationship between the two parameters (OS and PFS) and tumor-SVZ distance in patients with non-SVZ-contacting glioma. Combination analysis of the tumor-SVZ distance and IDH status showed that IDH1 mutation and SVZ non-involvement enable favorable outcomes, whereas IDH1 wild type with SVZ involvement indicates a significantly worse prognosis in all patients. Moreover, in patients with non-SVZ-contacting glioma, IDH1 mutation concurrent with tumor-SVZ distance >10 mm has better OS and PFS. IDH1 wild type and tumor-SVZ distance from 0 to 10 mm suggest poorer OS and PFS. Multivariate analysis showed WHO grade IV, SVZ involvement, tumor-SVZ distance from 0 to 10 mm, IDH1 mutation, gross total resection, and chemotherapy serve as independent predictors of OS. WHO grade IV, SVZ involvement, tumor-SVZ distance from 0 to 10 mm, IDH1 mutation, and chemotherapy serve as independent predictors of PFS of patients with glioma. In conclusion, tumor-SVZ distance and IDH1 mutation status are the determinants affecting patient outcome.

## Introduction

Glioma is the most common type of tumor in the brain, representing 75% of primary brain tumors in adults (1). Glioblastoma (GBM), as the most common aggressive cancer of the central nervous system, represents 45% of gliomas (2). GBM presents with the poorest prognosis and frequent relapses and is resistant to conventional therapies, including chemotherapy and radiation (3), with a 5-year survival of around 5% (2). Despite therapeutic strategies that have been improved, there is no substantial improvement in the overall survival of glioma (especially GBM) over the past decade, partially owing to the not-fully-understood mechanisms of glioma initiation and progression.

It has been proposed that glioma is initiated and propagated from a subpopulation of cancer cells with self-renewal ability, proliferative capacity, and multilineage potency, which are termed as glioma stem cells (GSCs) (4, 5). Converging evidence over the last decade has demonstrated that GSCs may arise from neural stem cells (NSCs) residing in the adult subventricular zone (SVZ) (6). This notion has been supported by the direct genetic evidence that astrocyte-like NSCs in the SVZ harbor the driver mutations of human glioma, which could lead to glioma development (6–11). These findings established a clinical link between NSCs in the SVZ and the initiation and progression of the glioma, and possible therapeutic interventions to improve outcomes of glioma.

Despite the critical roles of SVZ in the tumorigenesis and progression of glioma, the association of SVZ with clinical characteristics of glioma patients remains incompletely clarified. Recent studies have demonstrated that patients with glioma involving the SVZ are more likely to present multifocal lesions on MR images when initially diagnosed (12) and are associated with higher risk of recurrence and shorter overall survival (OS) (13–17). In addition, a shorter distance from the tumor centroid to the SVZ (≤30 mm) was correlated with unfavorable clinical outcome in patients with SVZ contact (17). However, many gliomas located in the parenchyma without SVZ contact are close to the SVZ. There is a lack of evidence with respect to the clinical correlation of the shortest distance from tumor edge to SVZ with prognostic consequence of patients with glioma.

While isocitrate dehydrogenase 1 (IDH1) mutations are present in 70% of lower-grade gliomas and secondary glioblastomas (18–20), and predict better patient survival, whether combined IDH1 mutation status and anatomical tumor-SVZ distance would facilitate more accurate prediction of prognosis has not yet been investigated. In the present study, we aim to assess the relationship of combined IDH1 mutation status and anatomical tumor-SVZ distance to clinical features of patients with glioma.

## Materials and Methonds

### Patients

We performed a retrospective investigation of patients who were diagnosed with glioma at our institution between 2010 and 2016. One hundred and forty-six patients who met the following criteria were enrolled: histopathological confirmation of glioma (WHO classification II–IV), underwent radiation and/or chemotherapy post surgical resection without prior craniotomy or stereotactic biopsy. All patients underwent preoperative and postoperative enhanced MRI scan and genetic detection of IDH1 mutation status. The shortest distance between the tumor edge and lateral ventricles was determined according to the preoperative MR (T2) images.^13^ Based on the distance=0 (SVZ involvement) and distance=10 mm (the median distance of all tumors that did not contact the ventricle) (16), patients were categorized into three groups according to the following criteria: type I (SVZ involvement), type II (tumor-SVZ distance from 0 to 10 mm), and type III (tumor-SVZ distance >10 mm). The extent of surgical resection was defined by comparing the preoperative and postoperative MR images. The gross total resection (GTR) was achieved by complete resection of the contrast-enhancing tumor on T1-weighted sequences. Otherwise, < GTR was defined as any resection that failed to achieve GTR (21). The tumor size and edema volume were determined by preoperative MR images (T2/fluid attenuation inversion recovery, T2 weighted image). Other clinical variables such as age, sex, and preoperative KPS score were obtained from medical records. All manipulations were performed under the approval of the Research Ethics Committee of the First Hospital Affiliated to Army Medical University (Southwest Hospital), and written informed consent was obtained from all patients.

### Magnetic Resonance Imaging and Data Processing

All patients underwent the same MRI protocol. MR images were acquired using conventional spin echo sequences on a 3.0-Tesla Siemens MR Magnetron System (Siemens AG, Erlangen, Germany) in axial plane, sagittal section, and coronal section. Patients received intravenous contrast (gadolinium diethylenetriamine pentaacetate) at a dose of 0.1 mmol/kg body weight. The MRI slice thickness was 5 mm.

**Figure 1:**
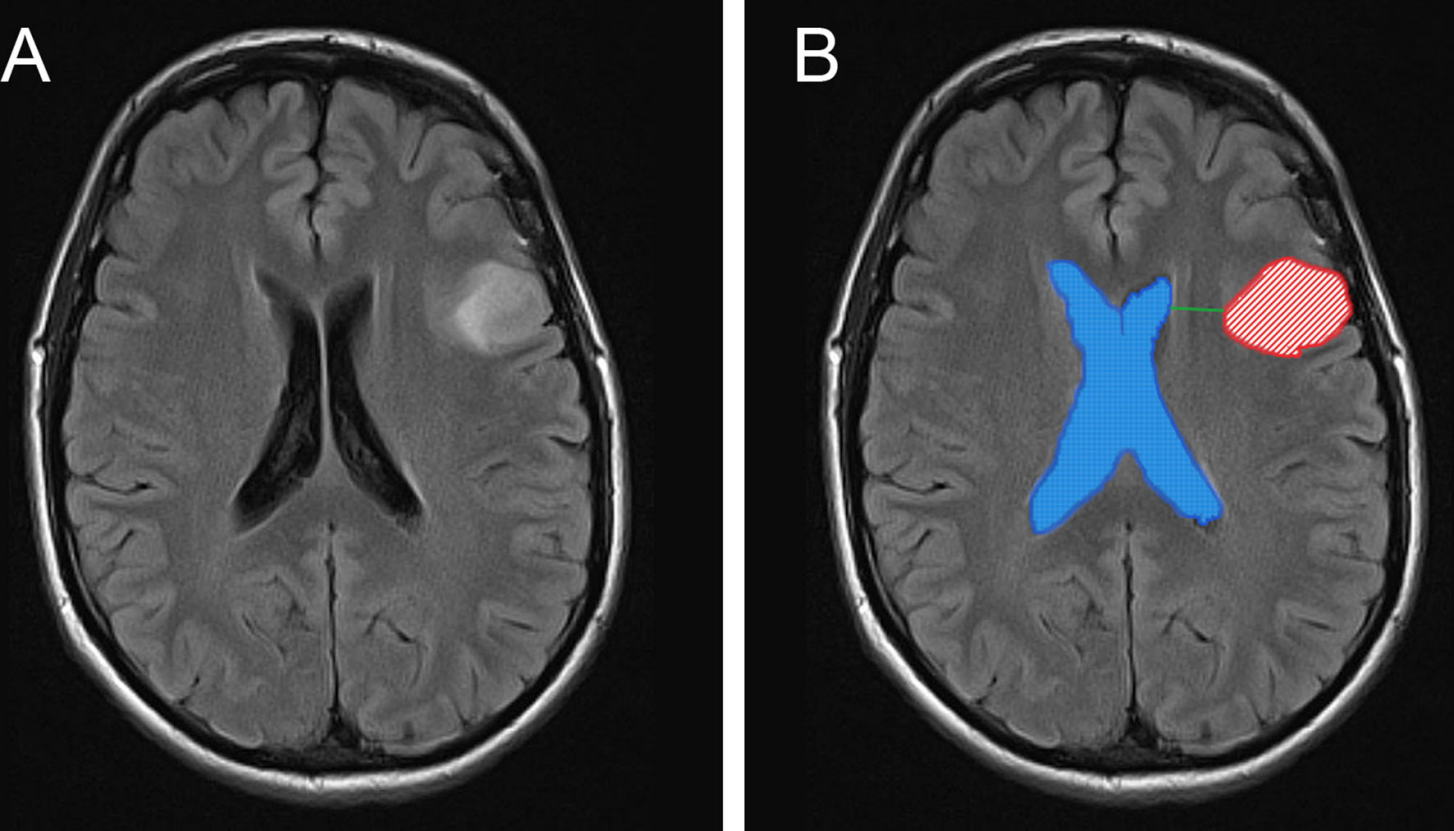
MR images depicts the anatomical position between tumors and the SVZ, calculation methods of V_G_ and V_GW_. Representative MR image of tumor without SVZ involvement **(A)**. The tumor lesions and ventricles are manually segmented and shown as red and blue masks. The shortest distance between the tumor edge and the lateral ventricles was showed as green line **(B)**.

For imaging analysis, tumors lesions and the lateral ventricle were segmented manually (**Figure 1B**) by two experienced neurosurgeons (more than 10 years of work experience) using INFINITT PACS software and 3D slicer (Surgical Planning Laboratory, version 4.10.2, Harvard University, Boston, MA, USA), and further reviewed by two independent neuroradiologists; they were blinded to outcomes for subsequent analysis. In both software packages, tumor segmentation was performed by delineating the abnormal signal in T2/fluid attenuation inversion recovery (T2/FLAIR) images (**Figure 1A**) (22). Surfaces representing the SVZ were created manually based on the description given by Vescovi et al. (23) and the rendering of the lateral ventricles given in the Hammersmith atlas (24). The volume of gross tumors (VG) and the volume of gross whole (VGW) are calculated by 3D slicer (**Supplementary Figure 1**); the workflow for this software has previously been described by others (25). The tumor-SVZ distance was defined as the shortest distance between the edge of tumor and the ependyma of the ventricles (13) and calculated by INFINITT PACS software (**Figure 1B**).

### Postoperative Outcomes

The clinical records of the included patients were gathered. OS was measured from the date of initial surgery to the date of death or date of last contact if alive. Progression-free survival (PFS) was calculated from the date of the primary surgical resection to disease progression or the last recorded date of follow-up without progression. All patients with disease progression were determined based on MR images, and disease progression was defined as a new or progressive increase within the initial surgical resection site and/or in a remote location, presenting symptoms or postoperative neurological dysfunction by a neurosurgeon and a radiologist blinded to outcomes.

### Statistical Analysis

Data were summarized as categorical variables, and differences between groups were tested using the Chi-square test. All collected data were statistically analyzed in SPSS version 23.1 (IBM Corp, Armonk, NY, USA). OS and PFS were plotted using the Kaplan-Meier method, and log-rank test was used to compare Kaplan-Meier plots. For patients without SVZ involvement, simple linear regression was used to determine the strength of association between the tumor-SVZ distance and the OS, PFS ​as indicated by Pearson’s correlation coefficients (GraphPad Prism 7, La Jolla, CA, USA). A *p* < 0.05 was considered to be statistically significant, and two-sided statistical tests were performed. Multivariate proportional hazards regression (Cox) analysis was used to analyze an association of independent factors with survival and recurrence. We performed both univariate and multivariate analyses. Factors with a probability value (*p*) < 0.05 on univariate analyses were subjected to multivariate Cox regression analyses.

## Results

### Patient Population and General Characteristics

One hundred and forty-six cases who met the inclusion criteria were retrospectively reviewed. Detailed clinical information is summarized in **Table 1**. Of the enrolled patients, 72 cases were found to be SVZ involved, which was grouped as type I. For type II (tumor-SVZ distance from 0 to 10 mm), 36 patients were selected. Thirty-eight cases were grouped as type III (tumor-SVZ distance >10 mm). In all patients, tumors and edemas were bigger in patients with SVZ-positive tumor than in patients with SVZ-negative tumor. A lower rate of GTR and low-grade gliomas (WHO II) was observed in patients with SVZ involvement (20.8 and 40.3%) than in patients with SVZ non-involvement (50 and 61.8%). The comparison in all patients indicates a lower IDH1-mutation ratio in patients with SVZ involvement (41.7%) than in patients with SVZ non-involvement (61.8%). Among the 146 patients included, 63 recurrent cases were observed, and 54 patients had died. The median follow-up time was 1,576 days at the time of last update during follow-up.

**Table 1:** Clinical characteristics (n = 146).

variables	SVZ(+)		SVZ(−)		
Tumor-SVZ distance (mm)	Type I^a^	p*^d^	Type II^b^	Type III^c^	p*^e^
**Number (%)**	72		36	38	
Age (≥40 Y, %)	40 (55.6)	0.98	22 (61.1)	19 (50.0)	0.33
Sex (man, %)	40 (55.6)	0.72	24 (66.7)	15 (39.5)	**0.01**
KPS <80 (%)	10 (13.9)	0.15	3 (8.3)	2 (5.3)	0.59
Volume >30 cm^3^ (%)	44 (61.1)	**0.001**	12 (33.3)	8 (21.1)	0.23
Edema >100 cm^3^ (%)	44 (61.1)	**0.001**	10 (27.8)	8 (21.1)	0.50
Side (Left, %)	38 (52.8)	0.61	13 (36.1)	23 (60.5)	**0.03**
GTR (%)	15 (20.8)	**0.001**	15 (41.7)	23 (60.5)	0.10
Radiation therapy (%)	39 (54.2)	0.73	19 (52.8)	19 (50.0)	0.81
Chemotherapy (%)	38 (52.8)	0.06	23 (63.9)	28 (73.7)	0.36
IDH1 mutation (%)	30 (41.7)	**0.008**	26 (72.2)	21 (55.3)	0.13
**Tumor location**					
Frontal lobe (%)	37 (51.4)		19 (52.8)	15 (39.5)	
Temporal lobe (%)	13 (17.3)	0.24	10 (27.8)	12 (31.6)	0.47
Others (%)	22 (19.7)		7 (8.8)	11 (28.9)	
**Regions contacting SVZ**		0.13			
Frontal horn (%)	33 (45.8)				
Temporal horn (%)	16 (22.2)				
Body (%)	12 (16.7)				
Occipital horn (%)	11 (15.3)				
**Recurrence pattern**					
Non-recurrence (%)	33 (45.8)		22 (61.1)	28 (73.7)	
Local (%)	32 (44.4)	**0.01**	12 (33.3)	10 (11.3)	0.24
Distant (%)	7 (9.7)		2 (5.6)	0	
**WHO classification**					
2 (%)	29 (40.3)	**0.01**	24 (66.7)	23 (60.5)	0.85
3 (%)	21 (29.2)		7 (19.4)	9 (23.7)	
4 (%)	22 (30.6)		5 (13.9)	6 (15.8)	

GTR, gross total resection; KPS, Karnofsky Performance Status scale; SVZ, subventricular zone. Bold values mean statistically significant.

*Chi-square test.

^a^SVZ involvement.

^b^tumor-SVZ distance from 0 to 10 mm.

^c^tumor-SVZ distance >10 mm.

^d^Comparison between SVZ involvement tumors and SVZ non-involvement tumors.

^e^Comparison between SVZ non-involvement tumors with different tumor-SVZ distance.

### Survival Analysis

**Figure 2:**
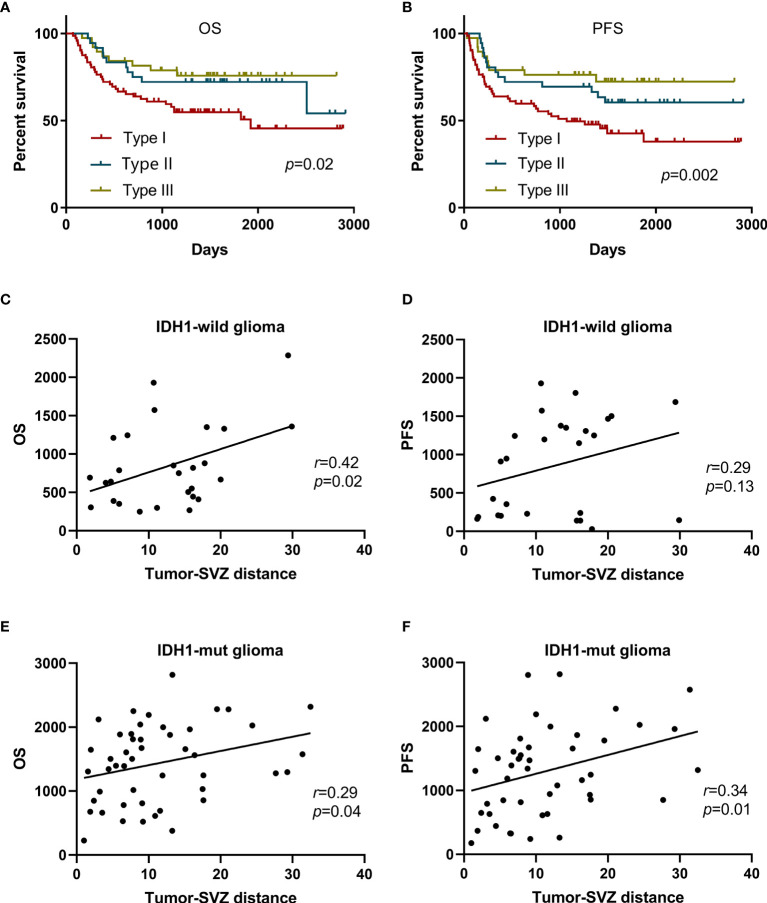
Kaplan-Meier curve analysis of all patients and simple linear correlation analysis of patients with non-SVZ-contacting glioma. Kaplan-Meier curves plotting of patient OS **(A)** and PFS **(B)** stratified by the tumor-SVZ distance. Simple linear correlation between the OS and tumor-SVZ distance in IDH1-wild glioma **(C)** and IDH1-mut glioma **(E)**. Simple linear correlation between the PFS and tumor-SVZ distance in IDH1-wild glioma **(D)** and IDH1-mut glioma **(F)**. Type I means SVZ involvement, type II means tumor-SVZ distance from 0 to 10 mm, type III means tumor-SVZ distance >10 mm.

Based on the tumor-SVZ distance stratification, the OS (p = 0.02) and PFS (p = 0.002) for the patients had a positive correlation with the tumor-SVZ distance. The patients with SVZ involvement had the worst OS and PFS. In striking contrast, tumor-SVZ distance >10 mm enabled favorable outcomes (**Figures 2A, B**). Simultaneously we investigated the relationship between the clinical outcomes (PFS and OS) and tumor-SVZ distance in low-grade gliomas (II) and high-grade gliomas (III-IV). Our results showed that tumor-SVZ distance >10 mm was better than tumor-SVZ distance from 0 to 10 mm in respect to OS in two groups, and tumor-SVZ distance >10 mm was associated with better PFS in low-grade gliomas (**Supplementary Figure 2**). Then, we divided the non-SVZ-contacting glioma into two groups: IDH1-wild glioma and IDH1-mut glioma. Simple linear correlation analysis was applied to investigate the relation between the two parameters (OS and PFS) and tumor-SVZ distance. The results revealed the positive correlation between the tumor-SVZ distance and two parameters (OS and PFS) except PFS of patients with IDH1-wild glioma (**Figures 2C–F**).

Furthermore, we performed comparison, taking into account tumor localization to the SVZ, IDH1 status, and WHO grade, respectively. In our cohort, patients with SVZ involvement were found to be associated with worse OS (*p* = 0.006) and PFS (*p* = 0.002) compared with those without SVZ contact (**Supplementary Figures 3A, B**), similar to previous studies (14, 17). IDH-mut glioma had better OS (*p* < 0.001) and PFS (*p* < 0.001) compared with patients without IDH1 mutation (**Supplementary Figures 3C, D**), which was in agreement with studies reported elsewhere (26, 27). The prognosis of patients with high WHO grade was worse than the patients with low WHO grade (**Supplementary Figures 3E, F**).

**Figure 3:**
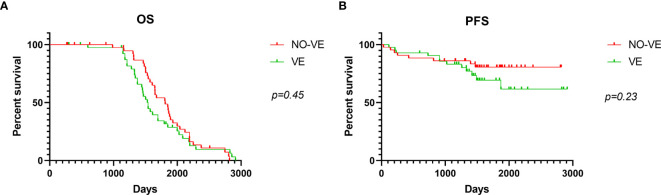
Kaplan-Meier curve analysis according to ventricular entry. OS **(A)** and PFS **(B)** analysis using Kaplan-Meier curves in patients with or without ventricular entry. VE means ventricular entry.

Considering the long-lasting debate of ventricular entry and possible dissemination, we analyzed the prognosis of low-grade patients (WHO grade II) according to the ventricular entry during surgical resections. Among the 76 low-grade gliomas (WHO II), there occurred 36 ventricular entries (VEs) during resection and 40 did not occur. There were 3 (8.3%) distant recurrences and 7 (19.4%) local recurrences in patients with ventricular entry. Whereas in patients with non-ventricular entry, no distant recurrence was observed and 8 (20%) patients had local recurrence. There was no statistically significant difference in local or distant recurrence by analysis of Chi-square test (*p* = 0.20). In addition, Kaplan-Meier analysis was used to perform survival analysis, and the differences were not statistically significant (**Figures 3A, B**).

### SVZ Contact and IDH1 Status

**Figure 4:**
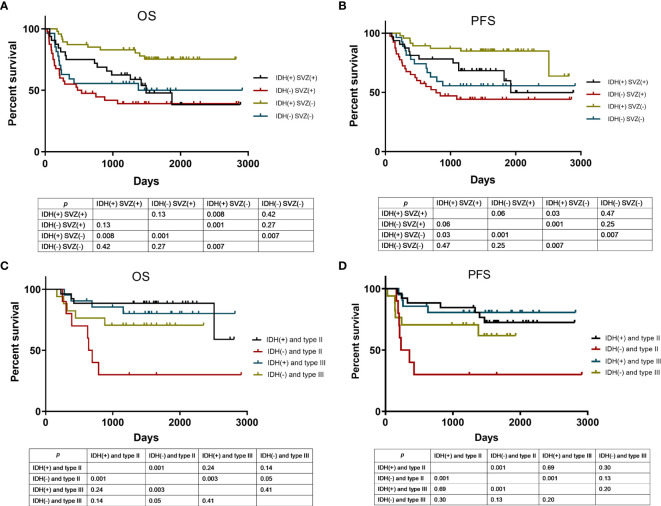
Kaplan-Meier curve analysis according to various risk categories. OS **(A)** and PFS **(B)** analysis using Kaplan-Meier curves in patients stratified by SVZ involvement and IDH1 mutation status. Of the patients without SVZ involvement, they were stratified by tumor-SVZ distance and IDH1 mutation status for OS **(C)** and PFS **(D)** analysis using Kaplan-Meier curves. SVZ (+) means SVZ involvement, SVZ (−) means SVZ non-involvement. IDH (+) means IDH1 mutation, IDH (−) means IDH1 wild type. Type II means tumor-SVZ distance from 0 to 10 mm; type III means tumor-SVZ distance >10 mm.

Given that SVZ contact and IDH1 mutation are potent factors predicting patient survival time and tumor relapse, it is unknown whether the prognostic performance would be improved with combined use of SVZ contact and IDH1 mutation. In patients with SVZ-positive tumors, no significant difference was found in PFS and OS between IDH1 mutation and IDH wild type. Among patients with SVZ-negative glioma, there was a positive association of IDH1 mutation with OS (*p* = 0.007) and PFS (*p* = 0.007) by contrast to wild-type IDH1. Importantly, both IDH1 mutation and SVZ-negative tumors enabled favorable patient outcomes and longer time to relapse, whereas IDH1-wild glioma with SVZ-positive displayed a significantly worse prognosis and shorter time to relapse (**Figures 4A, B**). These observations highlight the prognostic significance of combined detection of IDH1 status and SVZ involvement for patients with glioma.

### Tumor-SVZ Distance and IDH1 Status

As revealed above, in patients without SVZ involvement, the tumor-SVZ distance impacts the outcome of patients with glioma, among which patients with tumor-SVZ distance >10 mm have better prognosis than those with tumor-SVZ distance from 0 to 10 mm. To estimate the prognostic performance of combined tumor-SVZ distance and IDH1 status in patients without SVZ involvement, we made an additional comparison. As shown in **Figures 4C, D**, tumor-SVZ distance showed no obvious interaction with the OS and PFS of patients with IDH1 mutations. But in patients with IDH1 wild type, the OS (*p* = 0.05) of patients with tumor-SVZ distance >10 mm was significantly better than in patients with tumor-SVZ distance from 0 to 10 mm. Therefore, patients bearing wild-type IDH1 and tumor-SVZ distance from 0 to 10 mm displayed poorer OS and PFS. Whereas patients with IDH1 mutation concurrent with tumor-SVZ distance >10 mm exhibited particularly satisfactory prognosis and longer time to relapse.

### Univariate Analysis and Multivariate Analysis

The contribution of individual variables was evaluated by both univariate and multivariate analyses. Univariate analysis identifies unfavorable factors for the outcome of patients with glioma including age ≥40 years, KPS <80, WHO grades (III–IV) and SVZ involvement, and tumor-SVZ distance from 0 to 10 mm. GTR, IDH1 mutation, and chemotherapy were found to predict good prognosis of patients. A detailed summary is shown in **Table 2**. Similarly, multivariate analysis showed that WHO grade IV (*p* = 0.001), SVZ involvement (p = 0.01), tumor-SVZ distance from 0 to 10 mm (p = 0.03), GTR (p = 0.05), chemotherapy (p = 0.003), and IDH1 mutation (*p* = 0.01) served as independent predictors for patient OS. In addition, WHO grade IV (*p* = 0.001), SVZ involvement (p = 0.002), tumor-SVZ distance from 0 to 10 mm (p = 0.02), chemotherapy (p = 0.02), and IDH1 mutation (*p* = 0.03) served as independent predictors for patient PFS (**Table 3**).

**Table 2:** Univariate analysis of survival outcomes in all patients.

Characteristic	OS			PFS		
	*p*	HR	95% CI	*p*	HR	95% CI
Age ≥40 years	**0.01**	1.48	1.27–2.53	**0.01**	1.93	1.14–3.26
Sex (Male)	0.39	1.25	0.74–2.12	0.41	1.22	0.75–2.01
KPS <80 (preoperative)	**0.003**	2.72	1.40–5.27	**0.003**	2.68	1.39–5.16
Volume >30 cm^2^	0.91	1.02	0.61–1.73	0.57	1.15	0.70–1.88
Edema >100 cm^2^	0.29	1.21	0.37–1.82	0.52	1.17	0.71–1.92
Side (Left)	0.31	1.30	0.77–2.19	0.30	1.29	0.79–2.12
GTR	**0.003**	0.38	0.21–0.72	**0.018**	0.51	0.29–0.89
Radiation therapy	0.06	0.59	0.35–0.99	0.08	0.64	0.39–1.05
Chemotherapy	**0.001**	0.30	0.17–0.51	**0.001**	0.33	0.20–0.54
IDH1-mut	**0.001**	0.23	0.13–0.42	**0.001**	0.29	0.17–0.49
**Tumor location**	0.74			0.72		
Frontal lobe/others	0.56	0.83	0.44–1.55	0.66	0.88	0.50–1.55
Temporal lobe/others	0.90	1.04	0.52–2.08	0.41	0.74	0.36–1.51
**WHO**	**0.001**			**0.001**		
Grade 3/Grade 2	0.09	1.87	0.89–3.94	**0.008**	2.47	1.27–4.80
Grade 4/Grade 2	**0.001**	10.9	5.85–20.5	**0.001**	12.4	6.64–23.3
**Regions contacting SVZ**	0.73			0.28		
Frontal/Occipital	0.39	0.72	0.34–1.58	0.43	0.75	0.37–1.52
Temporal/Occipital	0.79	0.89	0.42–2	0.21	0.58	0.25–1.35
Body/Occipital	0.94	1.03	0.42–2.47	0.65	1.20	0.54–2.64
**Tumor-SVZ distance**	**0.004**			**0.002**		
Type I*^a/^*Type II^b^	**0.001**	4.25	1.79–10	**0.001**	5.31	2.10–13.4
Type II^b^/type III^c^	**0.02**	3.07	1.16–7.94	**0.004**	4.31	1.60–11.7

HR, Hazard Ratio; CI, Confidence Interval; GTR, Gross total resection; KPS, Karnofsky Performance Status scale; OS, Overall survival; PFS, progression-free survival outcomes; a, SVZ involvement; b, tumor-SVZ distance from 0 to10 mm; c, tumor-SVZ distance >10 mm; bold values mean statistically significant.

**Table 3:** Multivariate analysis of survival outcomes in all patients.

Characteristic	OS			PFS		
	*p*	HR	95% CI	*p*	HR	95% CI
Age ≥40 years	0.41	1.26	0.39–1.45	0.73	1.11	0.59–2.10
KPS <80	0.74	1.13	0.52–2.45	0.79	1.01	0.48–2.09
GTR	**0.05**	0.49	0.24–0.98	0.34	0.73	0.38–1.40
Chemotherapy	**0.003**	0.40	0.22–0.73	**0.02**	0.51	0.28–0.90
IDH1-mut	**0.01**	0.41	0.20–0.81	**0.03**	0.49	0.25–0.95
WHO	**0.001**			**0.001**		
Grade 3/Grade 2	0.3	1.50	0.65–3.45	0.11	1.83	0.86–3.87
Grade 4/Grade 2	**0.001**	8.94	4.03–19.8	**0.001**	8.89	4.07–19.4
**Tumor-SVZ distance**	**0.03**			**0.009**		
Type I*^a/^*Type II^b^	**0.01**	3.91	1.36–11.1	**0.002**	4.91	1.76–13.7
Type II^b^/type III^c^	**0.03**	1.34	1.06–7.71	**0.02**	3.29	1.20–9.01

HR, Hazard Ratio; CI, Confidence Interval; GTR, Gross total resection; KPS, Karnofsky Performance Status scale; OS, Overall survival; PFS, progression-free survival outcomes; a, SVZ involvement; b, tumor-SVZ distance from 0 to 10 mm; c, tumor-SVZ distance >10 mm; bold values mean statistically significant.

## Discussion

Our study demonstrates the relationship between the shortest distance from tumor edge to SVZ and the prognosis of patients with glioma. Although SVZ contact has been identified as a prognostic factor for outcome of patients with glioma, it remains undetermined whether and what distance from tumor to SVZ determine the prognosis of patients with glioma. We show that the clinical outcomes of patients with SVZ involvement were worse than patients with SVZ non-involvement regardless of IDH1 status. In patients with wild-type IDH1, the clinical outcomes of patients with tumor-SVZ distance from 0 to 10 mm were worse than those with tumor-SVZ distance >10 mm. Therefore, these data suggest that combining the distance from the tumor edge to the subventricular zone and IDH mutation provides a more accurate predictor for the prognosis of patients with Glioma.

Accumulating evidence indicates that SVZ and IDH1 mutation are the leading determinants for prognostic prediction of glioma. Studies over the last decade have established the critical role of SVZ in glioma development and progression. It is now considered that neural stem cells residing in the SVZ undergo neoplastic transformation and result in tumorigenesis, progression, and resistance to standard treatment (5, 11). Mutations in IDH1 occur in the vast majority of low-grade gliomas (WHO I–II) and secondary high-grade gliomas. Growing data indicate that these mutations drive gliomagenesis and are of prognostic importance for gliomas. However, little is known about whether combined IDH1 mutation and SVZ involvement are clinically relevant and provide improved prognostic performance for patients with glioma. Moreover, while its clinical role and significance in SVZ-positive patients were extensively studied, the functional consequence of tumor-SVZ distance in SVZ-negative patients has not been investigated. In this study, we performed volumetric analysis using MR images to reveal the anatomical relationship between the tumor progression and the distance from tumor edge to SVZ. SVZ involvement in our cohort is correlated with larger tumor and edema volumes, leading to lower rate of GTR. IDH1 mutation, which was found to disrupt SVZ and drive tumor progression (28), was detected in 53.2% of glioma population here, similar to prior studies (29, 30). We revealed the anatomical relationship between the tumor progression and the SVZ correlates with patient prognosis. However, further research is needed to conclusively determine the underlying mechanisms that have led to our results. Consistently, SVZ contact predicts patient outcomes described here and elsewhere regardless of IDH1 status. The observation that SVZ involvement was associated with shorter OS and PFS by univariate analysis is generally in line with multiple studies previously described (13, 14, 31, 32). Our findings here highlight the therapeutic implications of targeting SVZ for glioma intervention. Indeed, it has been shown that radiotherapy co-targeted to the SVZ improves PFS of patients compared to radiotherapy targeting the tumor alone (33). Additionally, increasing the mean dose of radiotherapy to the ipsilateral SVZ was associated with significantly improved OS (32, 34).

In addition, glioma, no matter the SVZ involvement or not, is thought to arise from SVZ where NSCs reside (5, 35–38). This means that non-SVZ-contacting glioma located at the parenchyma of the brain still has some correlation with SVZ, implying that the tumor-SVZ distance might affect the outcome of glioma. We evaluated the association between tumor-SVZ distance and the PFS and OS. Dramatically, our data suggest that tumor-SVZ distance was significantly associated with the PFS and OS of patients with glioma. Moreover, our data revealed the positive correlation between the two parameters (OS and PFS) and tumor-SVZ distance in patients with non-SVZ-contacting gliomas (**Figures 2C–F**). In multivariate analysis, SVZ involvement and tumor-SVZ distance from 0 to 10 mm serve as independent predictors for patients (**Table 3**). Thus, the present study supports that the tumor-SVZ distance correlates with the PFS and OS of glioma. This finding suggests that tumor-SVZ distance could be used as a new routine variable that can be used as a marker to predict the prognosis of patients, especially in patients without IDH mutation. The prognosis of tumors close to the SVZ may be worse than that of patients with tumors distant from the SVZ.

Despite the prognostic significance of SVZ involvement and IDH1 mutation status alone, evidence is lacking concerning their combined use. Mutations of the IDH1 are considered as an important event that occurs at an early stage during gliomagenesis (39) and was included in the (WHO) classification of the central nervous system published in 2016 (40). IDH1 mutations were thought to control the balance between glioma stem cell property and cell differentiation (41–45). A meta-analysis of 55 observational studies revealed glioma patients harboring IDH1 mutations have improved OS and PFS, especially for patients with WHO grade III and grades II–III (27, 46). We demonstrated in the current study that patients of IDH1 mutation concurrent with tumor-SVZ distance >10 mm have the best clinical outcome compared with patients bearing wild-type IDH1 and tumor-SVZ distance <10 mm, which exhibit the poorest survival time and shorter tumor relapse. It is interesting to clarify whether IDH1 mutation and tumor-SVZ distance >10 mm would serve as specific biomarkers that are predictive of effective response to current therapy for gliomas.

Our study also has limitations. This is a retrospective study, and its conclusion needs to be validated in future studies. Some established prognostic biomarkers, such as 1p/19q codeletion and MGMT promoter methylation status, are missing. Moreover, only the mutation status of IDH1 but not IDH2 was assessed here, although the IDH1 mutations were predominantly found (27). Additionally, current MR technology can hardly delineate the pathological boundary of a tumor lesion. A previous study pointed out that abnormality on T2WI represents a variable admixture of vasogenic edema and non-enhancing tumor, whereas abnormality on contrast-enhanced T1WI may correlate more closely with the site of origin of the glioma (13). Therefore, the edges of tumor are guided by standard MRI: T2WI for non-enhancing tumor in our study, which is consistent with the guideline of clinical treatment (47, 48). At the same time, to reduce inaccuracies, all tumor boundaries were manually defined by two experienced neurosurgeons and then reevaluated by two independent senior neuroradiologists. A prospective study designed for considering multiple variables might provide more accurate prognostic prediction for glioma.

## Data Availability Statement

The original contributions presented in the study are included in the article/**Supplementary Material**. Further inquiries can be directed to the corresponding authors.

## Ethics Statement

The studies involving human participants were reviewed and approved by the Research Ethics Committee of the First Hospital Affiliated to Army Medical University (Southwest Hospital). Written informed consent to participate in this study was provided by the participants’ legal guardian/next of kin.

## Author Contributions

RH and HF designed the experiments. SZ, DL, WF, XY, ZPL, YL, SH, YY, LT, WL, and FL collected the data. SZ, DL, FZ, and TZ evaluated the dataset and performed statistical analyses. CLa, ZL, CLi, and HL reviewed the radiological imaging. SZ, TZ, FZ, and RH drafted the manuscript. All authors contributed to the article and approved the submitted version.

## Funding

This work was supported by grants WSS-2014-11 from the Science and Technology Foundation of China Academy of Endineering Physics (Intersection of Physics and Biomedicine).

## Conflict of Interest

The authors declare that the research was conducted in the absence of any commercial or financial relationships that could be construed as a potential conflict of interest.

## Publisher’s Note

All claims expressed in this article are solely those of the authors and do not necessarily represent those of their affiliated organizations, or those of the publisher, the editors and the reviewers. Any product that may be evaluated in this article, or claim that may be made by its manufacturer, is not guaranteed or endorsed by the publisher.
