# Supplementary material for: Combination of the Distance From Tumor Edge to Subventricular Zone and IDH Mutation Predicts Prognosis of Patients With Glioma
Source: Front Oncol. 2021 Aug 19;11:693693. doi: 10.3389/fonc.2021.693693 (PMC8417404; doi:10.3389/fonc.2021.693693)
Supplement: Supplementary file 1 [file DataSheet_1.docx]

Supplementary Material

## Supplementary Figures


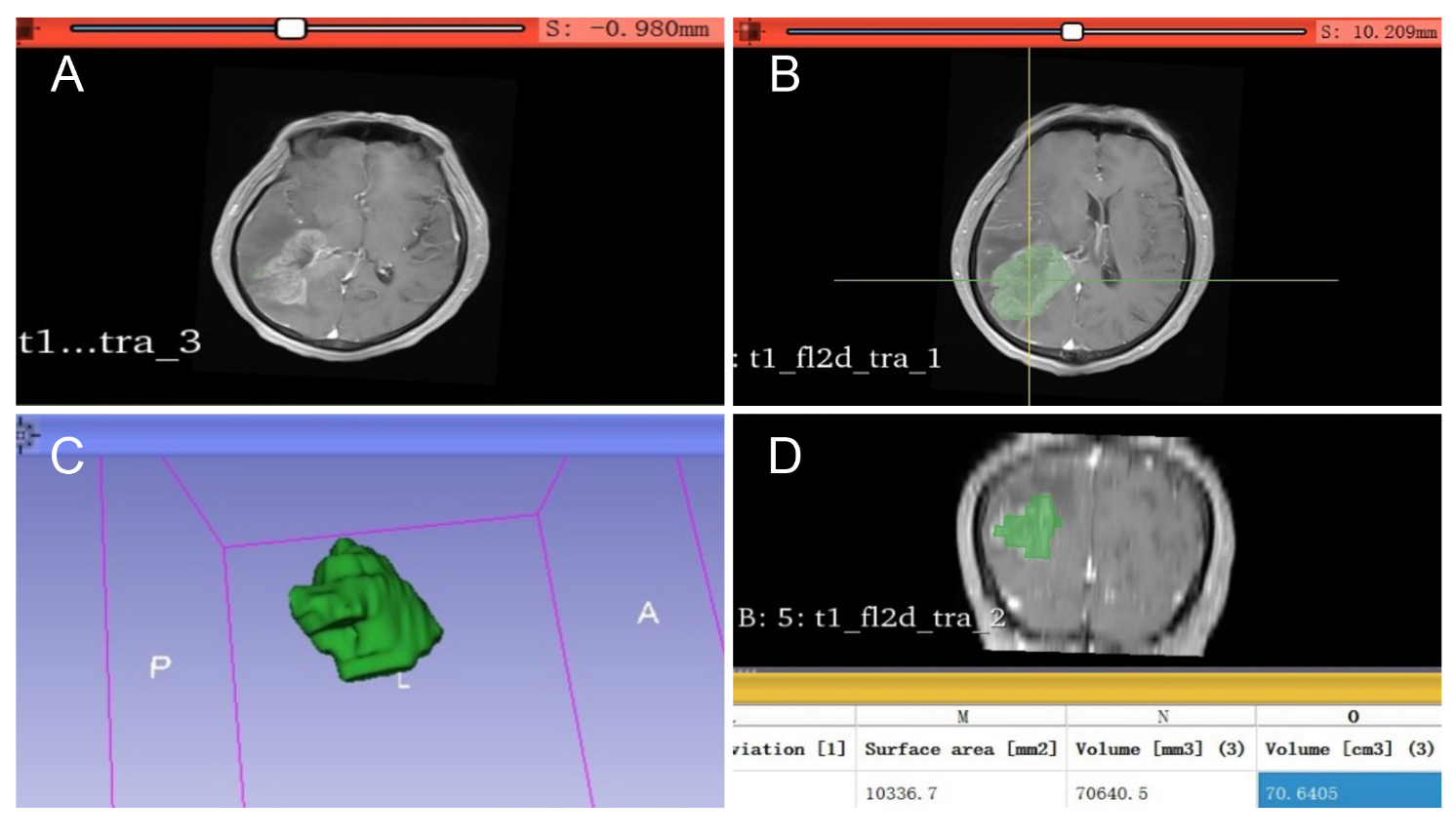


**Supplementary Figure 1.** (A) Import patient's image data (B) Draw the outline of the tumor or peritumoral edema (C) segment the tumor or peritumoral edema (D) calculate the volume of tumor or peritumoral edema.





**Supplementary Figure 2.** Of the patients without SVZ involvement, OS (A) and PFS (B) analysis using Kaplan-Meier curves in low-grade gliomas stratified by tumor-SVZ distance. OS (C) and PFS (D) analysis using Kaplan-Meier curves in high-grade gliomas stratified by tumor-SVZ distance. type II means tumor-SVZ distance from 0 to 10mm, type III means tumor-SVZ distance > 10mm.





**Supplementary Figure 3.** OS (A) and PFS (B) analysis using Kaplan-Meier curves in patients with or without SVZ contact. OS (C) and PFS (D) analysis using Kaplan-Meier curves in patients harboring IDH1 mutations or not. OS (E) and PFS (F) analysis using Kaplan-Meier curves in patients with different WHO grade (II vs II vs IV). SVZ (+) means SVZ involvement, SVZ (-) means SVZ no involvement. IDH (+) means IDH1-mutation, IDH (-) means IDH1-wild type.
